# Production and composition of extracellular polymeric substances by a unicellular strain and natural colonies of *Microcystis*: Impact of salinity and nutrient stress

**DOI:** 10.1111/1758-2229.13200

**Published:** 2023-09-11

**Authors:** Océane Reignier, Myriam Bormans, Laetitia Marchand, Corinne Sinquin, Zouher Amzil, Agata Zykwinska, Enora Briand

**Affiliations:** ^1^ PHYTOX, Laboratoire GENALG IFREMER Nantes France; ^2^ UMR CNRS 6553 ECOBIO University of Rennes 1 Rennes France; ^3^ MASAE, Laboratoire EM3B IFREMER Nantes France; ^4^ PHYTOX, Laboratoire METALG IFREMER Nantes France

## Abstract

The transfer of toxic cyanobacterial *Microcystis* blooms from freshwater to estuaries constitutes a serious environmental problem worldwide that is expected to expand in scale and intensity with anthropogenic and climate change. The formation and maintenance of *Microcystis* in colonial form is conditioned to the presence of extracellular polymeric substances (EPS). In this study, we attempted to better understand how the mucilaginous colonial form of *Microcystis* evolves under environmental stress conditions. In particular, we studied and compared the production and the composition of EPS fractions (attached and free) from natural colonies of a *Microcystis* bloom and from a unicellular *M. aeruginosa* strain under salinity and nutrient stress (representing a land‐sea continuum). Our results highlighted a greater production of EPS from the natural colonies of *Microcystis* than the unicellular one under nutrient and combined stress conditions dominated by the attached form. In comparison to the unicellular *Microcystis*, EPS produced by the colonial form were characterized by high molecular weight polysaccharides which were enriched in uronic acids and hexosamines, notably for the free fraction in response to increased salinities. This complex extracellular matrix gives the cells the ability to aggregate and allows the colonial cyanobacterial population to cope with osmotic shock.

## Graphical abstract

Our results highlighted a greater production of EPS from the natural colonies of *Microcystis* than the unicellular strain under nutrient and combined salinity stress conditions, dominated by the attached form. EPS produced by the colonial form were characterized by more diverse monosaccharides enriched in uronic acids and hexosamines. This complex extracellular matrix gives the cells the ability to aggregate and allows the colonial cyanobacterial population to better cope with an osmotic shock.

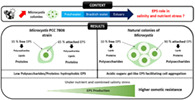

## INTRODUCTION

*Microcystis* is one of the most common toxic cyanobacterial genus able to proliferate worldwide in eutrophic freshwater environments (Preece et al., 2017). This genus forms colonies embedded in a mucilaginous matrix, and may proliferate into large floating biomass leading to ecological disturbances and representing a health risk for both humans and animals due to their capacity to produce toxins (Lance et al., 2010; Meriluoto et al., 2017; Metcalf et al., 2012). As nutrients and temperature are the main factors influencing cyanobacterial growth, the frequency, severity and duration of these mucilaginous *Microcystis* blooms are increasing due to eutrophication and climate change (O'Neil et al., 2012; Paerl et al., 2018; Preece et al., 2017; Rigosi et al., 2014).

The formation and maintenance of *Microcystis* in colonial form is conditioned to the presence of extracellular polymeric substances (EPS) (Chen et al., 2019; Xiao et al., 2019; Yang et al., 2008; Zhu et al., 2014). *Microcystis* EPS take the form of a mucus envelope sticking cells to each other known as mucilage. These EPS affect significantly the surface properties of the cells by electrostatic binding (Liu & Fang, 2002) or polymer bridging (Vogelaar et al., 2005) and consequently, have a strong impact on *Microcystis* aggregation and colony formation (Van Le et al., 2022).

Cyanobacterial EPS occur in different forms and, based on their degrees of adhesion cell (Zhou Yang et al., 2008; Qu et al., 2012), may be classified into free EPS (other names found in the literature are soluble macromolecules, slimes and colloids) and attached EPS (or named as sheaths, loosely bound polymers, transparent exopolymer particles (TEPs), capsular polymers, condensed gels and attached organic materials) (Laspidou & Rittmann, 2002; Nielsen & Jahn, 1999; Sheng et al., 2010). Free EPS are weakly bound with cells or dissolved into the environment, while attached EPS are closely bound with cells (Cruz et al., 2020; Delattre et al., 2016; Wingender et al., 1999). However, it should be mentioned that the definition of these two fractions (free and attached) is, in many studies, unclear and depends significantly on the chosen EPS extraction methods (Bourven et al., 2011; D'Abzac et al., 2010; Frølund et al., 1996). Comparisons of the available methods have been performed and different extraction strategies have been tested and developed, especially for *Microcystis* samples (Huang et al., 2021; Liu et al., 2014; Xu, Yu, et al., 2013). As an efficient and easy approach, low‐speed centrifugation or filtration recovering supernatant or the filtrate is widely accepted to extract free EPS (Huang et al., 2021; Pannard et al., 2016; Sheng et al., 2010). For attached EPS, extraction methods with various physical and chemical protocols like ultrasound‐assisted extraction or treatment with ethylenediaminetetraacetic acid (EDTA), cation exchange resin (CER), sodium hydroxide (NaOH) or formaldehyde have been proposed (D'Abzac et al., 2010; Liu & Fang, 2002; Sheng et al., 2005). More specifically for *Microcystis*,   Xu, Yu, et al. (2013) showed that the best method to isolate attached EPS was to heat the fixed cells at 60°C for 30 min in water.

Produced by different processes including excretion, sorption and cell lysis, EPS released by microorganisms are metabolic‐excess waste products (Fogg, 1983; Liu et al., 2018). Mague et al. (1980) determined that phytoplankton usually excrete 5%–20% of the carbon they fix which contribute to the nutrients and carbon pool, but more may be released under stress conditions (Fogg, 1983). The composition and production of EPS in cyanobacteria are both closely related to the taxonomy (Forni et al., 1997; Plude et al., 1991) and physiology (Xiao & Zheng, 2016) of the species/genus studied and are therefore strongly influenced by abiotic and biotic factors such as light (Li, Zhu, Gao, et al., 2013; Srivastava et al., 2019; Wei et al., 2021), temperature (Duan et al., 2018), nutrients (Wang, Liu, et al., 2011; Wang, Zhao, et al., 2011; Qu et al., 2018;), salinity (Ozturk & Aslim, 2010; Wang et al., 2013; Zhang et al., 2011), as well as predation (Cai et al., 2021; Yang et al., 2008). Among the above‐mentioned abiotic factors, salinity strongly affects the expansion of mucilaginous *Microcystis* blooms to estuaries and along the freshwater‐to‐marine continuum (Bormans et al., 2019; Paerl et al., 2018; Preece et al., 2017; Sampognaro et al., 2020). However, the production and composition of EPS associated with natural colonies of *Microcystis* under salt stress remain unknown.

Indeed, very few studies have focused on the characterization of the composition of both free and attached fractions of EPS in cyanobacteria and in particular in *Microcystis*. Moreover, these studies were conducted with *Microcystis* strains under laboratory conditions where *Microcystis* exist predominantly as single cells (Li, Zhu, Gao, et al., 2013; Yang et al., 2008;). Consequently, the ecological role of EPS fractions on natural *Microcystis* colonies under different environmental conditions remains unclear.

In the present study, the production and composition of EPS from a unicellular *Microcystis aeruginosa* strain (PCC 7806) were compared with those from mucilaginous natural colonies of a cyanobacterial bloom dominated by *Microcystis*. To get further insight into EPS composition and in particularly the presence of proteins, lipids and polysaccharides, free and attached fractions of EPS were prepared and analysed. In a second time, we explored whether EPS production from the unicellular and the mucilaginous colonial form of *Microcystis* was impacted when cells were exposed to a salinity stress. Finally, we investigated the EPS production of the natural colonies under a combined salinity and nutrient stress condition (representing a land‐sea continuum). We hypothesized that (1) EPS production would be higher for the mucilaginous colonial form than the unicellular form and (2) that the natural colonies would be more resistant to salt stress.

## EXPERIMENTAL PROCEDURES

### Strain and culture conditions

Isolated in the brackish water of Braakmann Reservoir in Netherlands, *M. aeruginosa* PCC 7806 strain from the Pasteur Culture (Paris, France) collection of Cyanobacteria (PCC; https://webext.pasteur.fr/cyanobacteria/) were routinely grown in modified BG11 (Rippka et al., 1979) supplemented with NaHCO_3_ (10 mM) and NaNO_3_ (2 mM), at constant temperature of 23°C under a 12:12 h light: dark cycle using cool‐white fluorescent tubes (Toshiba, 15 W, FL15D) with 30 μmol photons m^−2^ s^−1^ illumination. Following the protocol described in (Georges Des Aulnois et al., 2019), the PCC 7806 strain was acclimatized and routinely cultured at salinity 8. Under these conditions, PCC 7806 strain keeps its single‐cell form. This value of S = 8 was chosen in order to still obtain photosynthetically active/growing cells after 9 days of exposure but affecting EPS production from a stress condition. Salinity was checked using a conductivity meter Cond 3110 Set 1 (WTW, Oberbayern, Germany) and cultures were maintained in exponential growth phase by repeated dilution in fresh culture medium.

### *Natural colonies of* Microcystis *and batch experimentations*

#### Study site and sampling protocols

The Pen Mur freshwater reservoir (Morbihan, France) is located upstream the Saint Eloi River and the Pen Lan estuary (UTM easting: 538203.97, UTM Northing: 5267801.63). This site is used for the production of drinking water and it is subject to regular monitoring by the Regional Health Agency (ARS). This site was chosen because of the recurrent proliferation of cyanobacteria of the genus *Microcystis* (Bormans et al., 2019). During the summer of 2021, two sampling campaigns, corresponding to a phytoplankton bloom (September 6) and post‐bloom (September 21), were carried out. Ten litres of surface water samples (depth 0.25 m) were filtered with a 500 μm net, then brought directly to the laboratory in the dark conditions at 4°C, and used within hours of sampling for further analyses and batch experimentations.

#### Phytoplankton diversity

To quantify and analyse the phytoplankton diversity, we followed the standard protocol for sampling, conservation, observation and counting of lake phytoplankton for application of the Water Framework Directive (Version 3.3.1) (Laplace‐Treyture et al., 2009). Samples were fixed with acidic Lugol's solution (1% final concentration) and stored at 4°C until analysis. Species determination for cyanobacteria was based on morphological criteria using reference books (Bourelly, 1985; Komárek & Anagnostidis, 2008). Counts were performed at a magnification of ×320 with an inverted microscope (Zeiss Axio observer 5, Oberkochen, Germany). Photographs of the various taxa for each sample were taken to estimate their biovolumes and their reprocessing was done using the *Zen 2.3* software. Biovolumes were based on the geometrical estimation suggested by Sampognaro et al. (2020) for *Microcystis* and Sun and Liu (2003) for the other taxa.

#### Batch experimentations

To determine the effect of salinity and nutrient stress on the production and composition of free and attached EPS of natural *Microcystis* colonies, two batch experiments were performed. In the first experiment, called ‘Nutrient+’, we inoculated the bloom phytoplankton community in 2 L of modified BG11 medium with the salinity adjusted to 0, 5, 10 and 20 using artificial sea salt (Instant Ocean sea salt, Aquarium Systems) for 6 or 9 days. In the second experiment, called ‘Nutrient−’, we used the post‐bloom phytoplankton community as an inoculum in 2 L of Pen Mur reservoir water filtered through 0.2 μm without addition of nutrient to test the combined effect of salinity and nutrient limitation. The cultures were exposed during 6 days at salinities of 15 and 20, and 9 days at salinities of 0, 5 and 10. For the batch experiments, the upper salinity value tested was set up to S = 20, based on a recent study demonstrating a salinity tolerance of colonial strains of *M. aeruginosa* up to S = 20 (Bormans et al., 2023). For both batch experiments, the volume of inoculum was adjusted to obtain an initial *Microcystis* cell concentration of 10^5^ cells mL^−1^. All treatments were run in triplicate and performed in 4 L Erlenmeyer flasks under the same culture conditions as described above. Each flask was gently shacked manually twice a day, and their position in the incubation chamber was randomly changed every day.

### Nutrients

Water samples were filtered on GF/F filters (Whatman) and dissolved nutrient concentrations (phosphate and nitrate) were measured according to common colorimetric methods (Aminot & Chaussepied, 1983) with a sequential Gallery analyser (Thermo Fisher). Phosphate was measured with the method of Murphy and Riley (1962) and nitrate was measured after reduction to nitrite on a cadmium‐copper column (Henriksen & Selmer‐Olsen, 1970).

### EPS determination: free and attached EPS analyses

To characterize EPS from PCC 7806 strain and natural *Microcystis* colonies, 1.5 L of an exponential growing culture and 1–3 L of field sampling was centrifuged at 4000*g* during 30 min at 4°C. Supernatants, corresponding to free EPS, were concentrated and desalted using an ultra‐filtration system (Pellicon 2, Millipore). A 5 kDa cut‐off cassette was mounted to retain in the retentate molecules larger than 5 kDa such as proteins or polysaccharides, while smaller molecules, including salts, passed through the membrane and were eliminated in the filtrate. An additional water dialysis step for 3 days (3.5 kDa cut‐off porous membrane) was applied to remove the remaining salts (conductivity <50 μS cm^−1^), and then, samples were freeze‐dried.

Pellets, containing attached EPS with cells and debris, were fixed for 4 h at room temperature with a solution of 5% formol/ethanol (w/w) to prevent cell lysis, and dialysed against water for 3 days (3.5 kDa cut‐off porous membrane) to eliminate salts (conductivity <50 μS cm^−1^) and freeze‐dried. In a second step, to recover attached EPS eventually associated with *Microcystis* cells, pellets were dispersed and mixed gently in water for 1 h at 60°C (Roux et al., 2021;   Xu, Yu, et al., 2013). The heat could help to detach the EPS from the cells or other particulate matter in the pellet. After this process, samples were centrifuged during 15 min at 4000*g* to remove any cells, debris or insoluble material, and the resulting supernatant was freeze‐died for further analysis on attached EPS. A microscopic quality control step was performed to verify that more than 98% of the cells were not deformed/damaged/lysed in the sample to ensure the integrity of the cells retaining the intracellular content.

For EPS analyses, all samples were solubilized in water at 4 mg mL^−1^. To quantify protein concentration in EPS, the bicinchoninic‐acid protein assay (BCA), with Bovine Serum Albumin as standard, was used (Smith et al., 1985). The lipid content was estimated using the sulfo‐phospho‐vanillin (SPV) dosage (Frings et al., 1972), based on the method originally described by Bligh and Dyer (1959) with some modifications made by Axelsson and Gentili (2014), with virgin olive oil as internal standard.

Monosaccharide composition of free and attached EPS was determined according to Kamerling et al. (1975) method, modified by Montreuil et al. (1986). Samples were firstly hydrolyzed using MeOH/HCl 3 N at 100°C for 4 h. Myo‐inositol was used as internal standard. The methyl glycosides thus obtained were then converted to trimethylsilyl derivatives using *N*,*O*‐bis(trimethylsilyl)trifluoroacetamide and trimethylchlorosilane (BSTFA:TMCS) 99:1 (v:v). Gas chromatography with flame ionization detection (GC‐FID, Agilent Technologies 6890N) was used for separation, identification and quantification of the per‐*O*‐trimethylsilyl methyl glycosides formed. For better visualization, the weight percentages of monosaccharides have been recalculated from the total polysaccharide weight.

The molecular weights of polysaccharides were determined using High Performance Size‐Exclusion Chromatography (HPSEC, Prominence Shimadzu Co, Japan) composed of a Prominence Shimadzu HPLC system, a PL aquagel‐OH mixed column, an 8 μm (Varian) guard column (*U* 7.5 mm × *L* 50 mm), and a PL aquagel‐OH mixed (Varian) separation column. The HPSEC system was coupled on‐line with a multiangle light scattering detector (MALS) from Wyatt Technology (Dawn Heleos‐II, USA), a differential refractive index detector (RI) (Optilab Wyatt Technology) and a UV detector set to measure absorbance at *λ* = 280 nm. Samples were eluted with 0.1 M ammonium acetate at 1 mL min^−1^ flow rate. The molecular weight was calculated using a refractive index increment dn/dc of 0.145 used for polysaccharides. After obtaining the data from the HPSEC system, the chromatograms were further processed using *Astra 6.1* Software (Wyatt Technology).

## RESULTS AND DISCUSSION

### Physico‐chemical characteristics of Pen Mur reservoir and its phytoplankton/cyanobacteria community

The Pen Mur reservoir was a freshwater environment (S = 0.13 and 0.12, respectively at bloom and post‐bloom sampling dates; Table 1). The bloom sample was characterized by higher turbidity, temperature, chlorophyll *a* and nutrient concentrations than the post‐bloom sample (Table 1). While the bloom sample was associated with non‐limiting nutrients concentrations (P‐PO_4_ = 0.14 mg L^−1^ and N‐NO_3_ = 2.14 mg L^−1^, Table 1), the post‐bloom sample displayed strong phosphate limitation (P‐PO_4_ below quantification limit of 0.01 mg L^−1^, N‐NO_3_ = 2.31 mg L^−1^, Table 1).

**TABLE 1:** Physico‐chemical characteristics of the Pen Mur reservoir during bloom and post‐bloom sampling.

Sampling date	Time	Salinity	Water temperature (°C)	pH	Chl*a* (μg/L)	Turbidity (FNU)	N‐NO_3_ (mg L^−1^)	P‐PO_4_ (mg L^−1^)
06 September 2021	Bloom	0.13	24.2	9.71	1321.65	831.00	2.14	0.14
21 September 2021	Post‐bloom	0.12	19.2	8.31	50.73	16.10	2.31	<0.01

**FIGURE 1:**
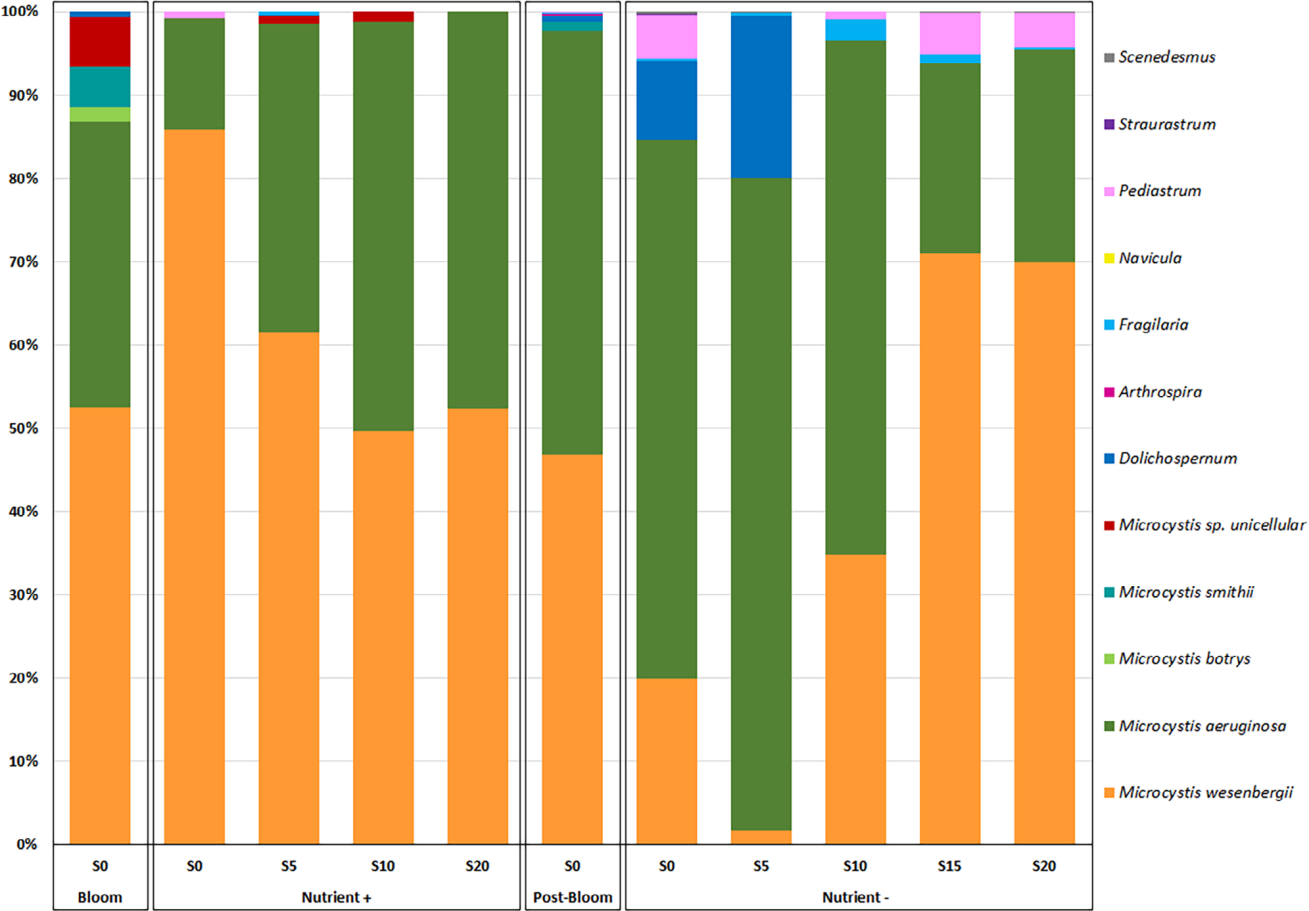
Phytoplankton community sampled in Pen Mur freshwater reservoir at bloom and post‐bloom and at the end of the two batch experiments (‘Nutrient+’ and ‘Nutrient−’) at salinity S = 0, S = 5, S = 10, S = 15 and S = 20.

The phytoplankton community sampled at the Pen Mur reservoir at both bloom and post‐bloom was strongly dominated by cyanobacteria (over 90% in biovolume; Figure 1). The cyanobacterial biomass was characterized by several species of *Microcystis* (*M. aeruginosa*, *M. wesenbergii*, *M. botrys*, *M. smithii* and *Microcystis* sp. unicellular) with *M. aeruginosa* and *M. wesenbergii* accounting for a minimum of 87% of the total biovolume. Hence, the content and composition of free and attached EPS described hereafter were mainly associated with a cyanobacterial community dominated by *Microcystis* genus. More specifically, the bloom sample displayed a higher cyanobacterial diversity than the post‐bloom sample.

### EPS yield and composition at salinity zero

The composition and quantification of free and attached EPS in natural bloom‐forming *Microcystis* colonies and in isolated strains may provide clues towards a better understanding of the mechanisms of cyanobacterial blooming and their ecological success.

#### EPS yields

**FIGURE 2:**
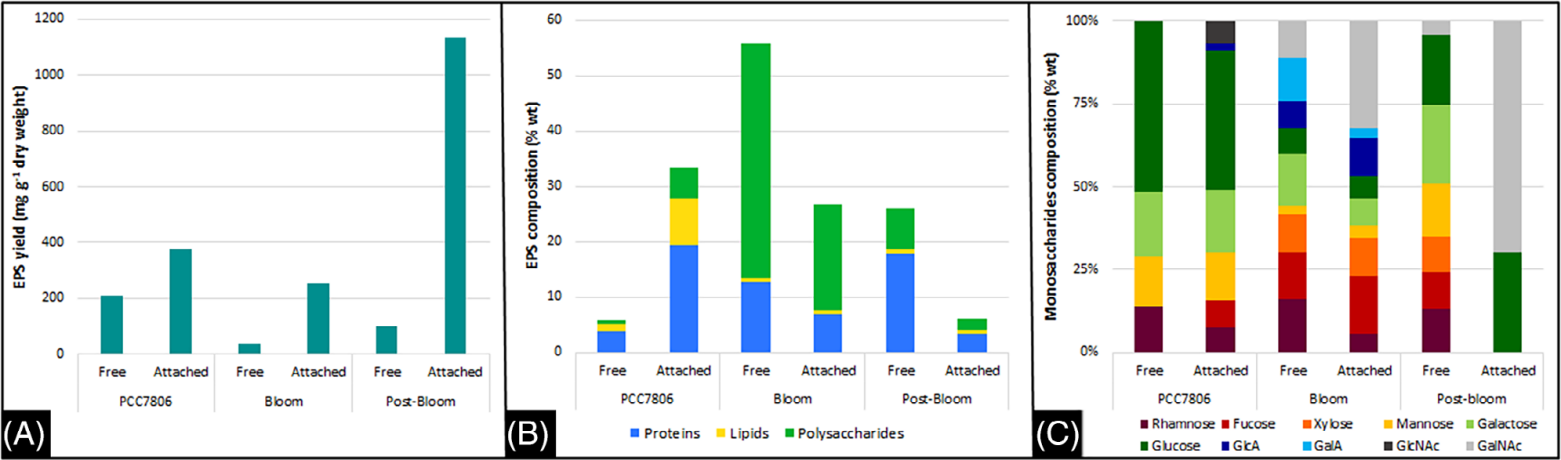
Free and attached EPS (A) yield, (B) proteins, lipids and polysaccharides composition and (C) monosaccharide composition of the polysaccharide fraction from the unicellular *M. aeruginosa* PCC 7806 strain and natural colonies of *Microcystis* during bloom and post‐bloom at salinity zero.

Under our culture conditions, total (free and attached) EPS yield for the unicellular PCC 7806 strain was 590 mg g^−1^ dry weight, corresponding to ~3 pg cell^−1^ (Figure 2A). The highest EPS yield was obtained in natural *Microcystis* colonies during post‐bloom (under nutrient limitation) than during bloom stage, with a total EPS production of 1236 and 290 mg g^−1^ dry weight, respectively (equivalent to ~10 and 5 pg cell^−1^). EPS production is known to increase under stress conditions including nutrient limitation (Ma et al., 2014) and more generally under conditions leading to lower specific growth rates (Li, Zhu, Gao, et al., 2013), which characterize the physiological state of cells during the post‐bloom period. Similar ranges of total EPS (between 1.5 and 10 pg cell^−1^) were reported by Xu, Cai, et al. (2013) and Xu, Yu, et al. for unicellular *M. aeruginosa*, by Duan et al. (2018) for attached EPS of colonial *M. wesenbergii* (3–7 pg cell^−1^) and by Chen et al. (2019) for soluble EPS for colonial *M. aeruginosa* (300–1000 mg g^−1^ dry weight). The amount of EPS produced was greater in the attached fraction than in the free fraction regardless of the sample, with the attached fraction accounting for 64%, 87% and 94% of the total EPS, respectively for PCC 7806 and colonies during bloom and post‐bloom. Higher yields of attached EPS than free EPS were also observed in the field‐sampled *Microcystis* colonies, whereas that of unicellular *Microcystis* PCC 7820 strain culture almost uniformly contained both fractions (Xu et al., 2014). Few studies investigated the role of EPS subfractions in the process of *Microcystis* aggregation and mucilaginous bloom formation. Some authors hypothesized that the proportion of EPS fractions for the field‐sampled *Microcystis* aggregates might influence the strength of adhesion between cells within the colony which may enhance the integrity of cell structure and protect the cells against biotic and abiotic stress conditions (Xu & Jiang, 2013; Xu, Cai, et al., 2013).

#### EPS composition

EPS are heterogeneous and complex materials primarily constituted of polysaccharides, proteins, lipids, nucleic acids and some inorganic components usually resulting from cell lysis and bacterial remineralization (Delattre et al., 2016; Liu et al., 2018). Here we measured the proportion of polysaccharides, proteins and lipids constituting the free and attached EPS (Figure 2B). Globally lipids are the minor components, representing less than 2% of the total EPS, except in the attached fraction of the PCC 7806 *M. aeruginosa* strain which represented 8%. Part of the lipid fraction measured may come from lipopolysaccharides (LPS), an important component of the outer membrane of cyanobacteria (Durai et al., 2015; Kehr & Dittmann, 2015). Proteins accounted for ~20% of the total EPS regardless of the sample but differences were observed in function of the fraction. A higher proportion of proteins in free EPS (~15%) than in attached EPS (~5%) was measured in the field samples, whereas the reverse was observed in the unicellular strain (~5% and ~20% in soluble and attached fractions, respectively). Proteins have a high binding strength (Sheng et al., 2010) that may increase cyanobacterial EPS firmness (Plude et al., 1991). Polysaccharides have been found as the major constituent (~ 60%) of total EPS in the natural *Microcystis* colonies under bloom condition, compared to 10% and 6% under post‐bloom stage and for the unicellular strain, respectively. Thicker polysaccharides envelope in colonial *M. aeruginosa* cells than the unicellular cells was already reported (Zhang et al., 2007). Known as the most complex biomacromolecules in biological systems, polysaccharides may also play a role in adhering cells together to form colonies (Yang et al., 2011), acting as physical barriers against different stress (Chen et al., 2019) and conferring to the mucilaginous colonial form of *Microcystis* the ability to cope with a multitude of environmental factors (Kehr & Dittmann, 2015).

To deepen the *Microcystis* polysaccharides characterization and to infer their physicochemical properties, the molecular weight of free and attached fractions were characterized by HPSEC‐MALS. In general, two main peaks were distinguished in field samples (bloom and post‐bloom), highlighting the presence of two polysaccharide populations (Figure S1A), respectively of high molecular weight (HMW) (from 1.4 × 10^6^ to 3.1 × 10^6^ g mol^−1^) and medium molecular weight (MMW) (from 4.6 × 10^4^ to 5.6 × 10^5^ g mol^−1^) (Figure S1B). Both polysaccharide populations were present in the similar proportion (mass fraction of 50%, Figure S1A). In the PCC 7806, HMW population was also observed in both free and attached EPS. However, a second HMW population, accounting for 25% of the total polysaccharide population, was only measured in the attached EPS, while the free EPS had slightly more than 50% of a low‐molecular weight (LMW) population (<5 × 10^4^ g mol^−1^), contrary to other samples. In the literature, exopolysaccharides of studied cyanobacteria species/genera are mainly characterized by high molecular weight (Pereira et al., 2009). However, these studies refereed to isolated strains but not on *Microcystis* colonies. The present study confirms that the natural *Microcystis* colony and PCC 7806 strain are mainly composed of high and medium molecular weight polysaccharides, and brings further inside in the characteristics of both free and attached polysaccharides. We also show here that natural colonies have higher proportions of high molecular weights than the unicellular strain, suggesting more complex biopolymers likely involved in colonies formation/cell aggregation.

#### Monosaccharides composition

Cyanobacterial polysaccharides are known as heteropolysaccharides consisting of complex repeating units, generally containing from 5 to 8 monosaccharides. The monosaccharide composition of polysaccharides constitutive of EPS produced by natural colonies of *Microcystis* is not well studied compared to EPS produced by isolated strains of *Microcystis* (De Philippis et al., 2001). Indeed, the exopolysaccharide composition from natural colonies is more variable and harder to predict, due to the influence of factors such as the surrounding environment and the presence of other microbial species. Additionally, the structural complexity of exopolysaccharides can make it difficult to isolate and purify the molecules for further study. Despite these challenges, studying the monosaccharide composition of the polysaccharides in EPS from natural colonies of *Microcystis* can provide valuable insights into the ecology and behaviour of these cyanobacteria in natural environments. Finally, the biotechnological potential of EPS from *Microcystis* has been recognized (Sun et al., 2015; Tan et al., 2023), and efforts are ongoing to develop methods for studying and exploiting these complex macromolecules (Duan et al., 2020). According to our experimental design, this study targeted the 10 most abundant and most commonly monosaccharides studied in the literature such as deoxyhexose (rhamnose, fucose), pentose (xylose), hexose (mannose, galactose, glucose), as well as acidic residues (glucuronic and galacturonic acids) and hexosamines (*N*‐acetyl‐glucosamine and *N*‐acetyl‐galactosamine) (Cruz et al., 2020; Li et al., 2001; Rossi & De Philippis, 2016).

By considering only the polysaccharide fraction presented in Figure 2B, the targeted monosaccharides found in the free EPS of PCC 7806 *M. aeruginosa* strain consisted of more than 85% of hexoses (50% of glucose, 20% of galactose and 15% of mannose; Figure 2C), and 15% of the deoxyhexose rhamnose, while no pentose or acid residues were detected. The major neutral sugars including rhamnose, mannose, galactose and glucose have been already reported for the PCC 7806 *M. aeruginosa* strain (Zhen Yang & Kong, 2012) and were also produced by other *Microcystis* species including *M. flos‐aquae* and *M. viridis* (Srivastava et al., 2019). However, some specificities have been highlighted depending on the species/strain studied and the growth conditions (Forni et al., 1997; Nakagawa et al., 1987; Plude et al., 1991). For example, fucose, arabinose and the disaccharide trehalose were species‐ or even strain‐specific of *Microcystis* (Forni et al., 1997).

In comparison, the monosaccharide composition of free EPS measured in bloom and post‐bloom samples was more diverse than that produced by the unicellular *M. aeruginosa* strain. Indeed, the natural colonies were dominated by two species, *M. aeruginosa* and *M. wesenbergii*. Independently of the period of the bloom, natural colonies of *Microcystis* produced polysaccharides composed of fucose, xylose, galacturonic and glucuronic acids and *N*‐acetyl‐galactosamine, in addition to the aforementioned hexoses and rhamnose retrieved also in the unicellular *Microcystis* strain. The composition of monosaccharides produced by natural *Microcystis* colonies during the bloom differed from those measured during the post‐bloom by their composition in uronic acids (presence of glucuronic and galacturonic acids only during the bloom). The presence of these acidic sugars has already been documented for cyanobacterial species (Cruz et al., 2020; Kehr & Dittmann, 2015). For example, uronic acids were found in EPS from different *Microcystis* species, including *M. wesenbergii* (Forni et al., 1997). Their anionic nature, allowing ionic interactions with positively charged macromolecules (e.g., proteins) and cations (e.g., calcium) present in water, would play a major role in aggregation of cyanobacterial EPS providing a ‘sticky’ or gel‐like behaviour to the overall macromolecule, further leading to the mucilaginous *Microcystis* bloom formation (Liu et al., 2018; Verspagen et al., 2006). Galacturonic acids were the main sugar measured in the EPS of *M. flos‐aquae* slime (Plude et al., 1991), and would affect the EPS solubility (Sutherland, 2001).

We further investigated the monosaccharide composition of attached EPS produced by *Microcystis*. Some studies reported that the monosaccharide composition of attached EPS in certain cyanobacteria (e.g., *Anabaena sphaerica* or *Fischerella musicola*) are remarkably different from those of free EPS (Forni et al., 1997; Gloaguen et al., 1995; Nicolaus et al., 1999). In our study, regardless of the samples, only slight changes in the monosaccharide composition between both fractions were observed, with lower amount of rhamnose and higher proportion of *N*‐acetyl‐galactosamine measured in the attached EPS compared to the free EPS. Notably, during bloom and post‐bloom, attached EPS were composed, respectively of 30% and 70% of *N*‐acetyl‐galactosamine. To our knowledge, this specific hexosamine was not described as a part of the monosaccharide composition of both *M. aeruginosa* EPS and LPS (Fujii et al., 2012; Martin et al., 1989). It was tempting to hypothesize that *M. wesenbergii*, which was one of the two dominant species in the natural cyanobacterial community, contributed to this composition. Indeed, it has been shown that free polysaccharide fraction in an isolated *M. wesenbergii* strain was exclusively composed of acid monosaccharides (Forni et al., 1997). Noteworthy, the composition and metabolic activity of the associated microbial community, notably heterotrophic bacteria, may also contribute to the observed variation of monosaccharide diversity in exopolysaccharides.

### Effect of a salinity shock associated or not with nutrient limitation

Several studies on the salinity tolerance of *Microcystis* have demonstrated highly variable results for salinity thresholds and tolerance for isolated unicellular strains in the range 7–14 (Georges Des Aulnois et al., 2020; Qiu et al., 2022; Ross et al., 2019; Tonk et al., 2007) for isolated colonial strains in the range 15–20 (Bormans et al., 2023) and for natural colonies in the range 10–17 (Kruk et al., 2017; Robson & Hamilton, 2003; Wang et al., 2022), highlighting strong intraspecific and interspecific variabilities, with higher salinity thresholds for colonial forms. As suggested by Kruk et al. (2017), the mucilage associated with the colonial form of *Microcystis* is likely to protect the cells from osmotic shock at high salinity. While the effects of salinity on *Microcystis* growth, toxin production and colony size have been well‐studied (Orr et al., 2004; Qiu et al., 2022; Ross et al., 2019; Sampognaro et al., 2020; Tonk et al., 2007), the effects of salinity on production and composition of *Microcystis* EPS are underexplored.

#### Phytoplankton/cyanobacterial assemblage

The phytoplankton communities sampled in the field at bloom and post‐bloom were used, respectively, as inoculum for the salt stress batch ‘Nutrient+’ and ‘Nutrient‐’ experiments (Figure 1). The ‘Nutrient+’ experiment was performed by inoculating the bloom phytoplankton community in BG11 medium in order to remain non‐limiting condition over the entire experiment. The resulting concentrations were P‐PO_4_ = 4.70 mg L^−1^ and N‐NO_3_ = 22.6 mg L^−1^ on day 0 and P‐PO_4_ > 3.5 mg L^−1^ and N‐NO_3_ > 15 mg L^−1^ on day 9. In the ‘Nutrient‐’ experiment, we used the post‐bloom phytoplankton community as an inoculum in the limiting‐nutrient Pen Mur water with no additional nutrients. The ‘Nutrient‐’ samples displayed strong phosphate limitation from day 0 (P‐PO_4_ below quantification limit of 0.01 mg L^−1^, N‐NO_3_ = 2.02 mg L^−1^) to day 9 (P‐PO_4_ < 0.01 mg L^−1^ and N‐NO_3_ > 0.87 mg L^−1^).

**FIGURE 3:**
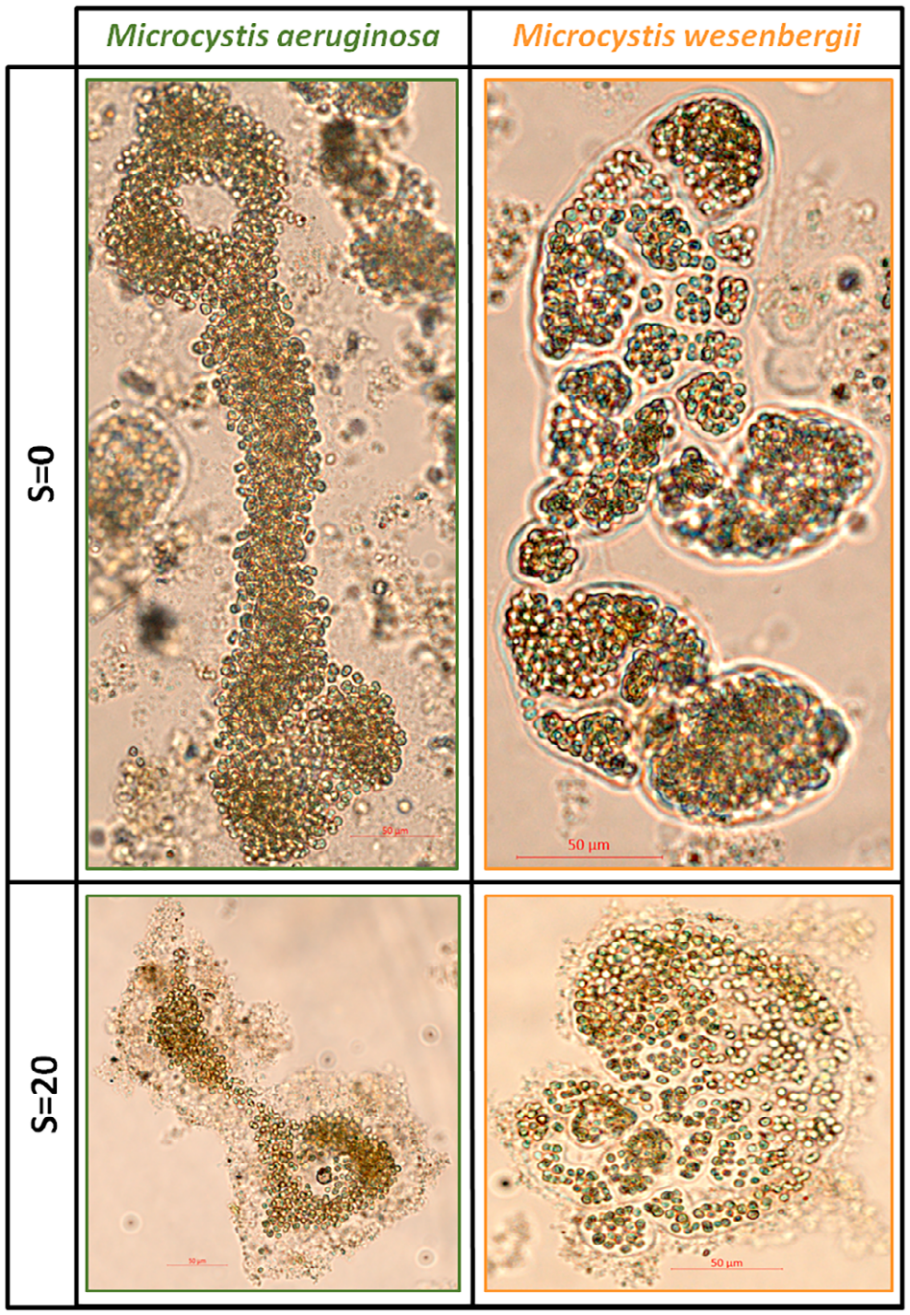
Microscopic photographs of a representative colony of *M. aeruginosa* and *M. wesenbergii* at salinity S = 0 and S = 20 showing the difference of thickness and boundary limit of the mucilage.

The phytoplanktonic diversity at the end of the batch ‘Nutrient+’ experiment (without nutrient limitation) was dominated by the genus *Microcystis* whatever the salinity (almost exclusively *M. wesenbergii* and *M. aeruginosa*). In particular, more than 50% of the biomass was associated with *M. wesenbergii* whatever the salinity. It is interesting to note that the proportion of *M. aeruginosa* increased at intermediate salinity values in accordance with previous studies demonstrating that natural colonies of *M. aeruginosa* have been reported to grow at salinity up to 15 (Kruk et al., 2017; Lehman et al., 2005). However, at higher salinity (S = 20), *M. wesenbergii* slightly dominated the cyanobacterial assemblage. Similarly, at the end of the batch ‘Nutrient‐’ experiment (with nutrient limitation), a higher salinity (S = 15 and 20) favoured *M. wesenbergii* over *M. aeruginosa*. To our knowledge, this high salinity tolerance of natural colonies of *M. wesenbergii* has never been reported before. We suggest that the thick, well‐defined mucilage associated with natural *M. wesenbergii* colonies, as observed in this study (Figure 3), is likely to act as strong physical barrier against increased salinity.

#### EPS yield

**FIGURE 4:**
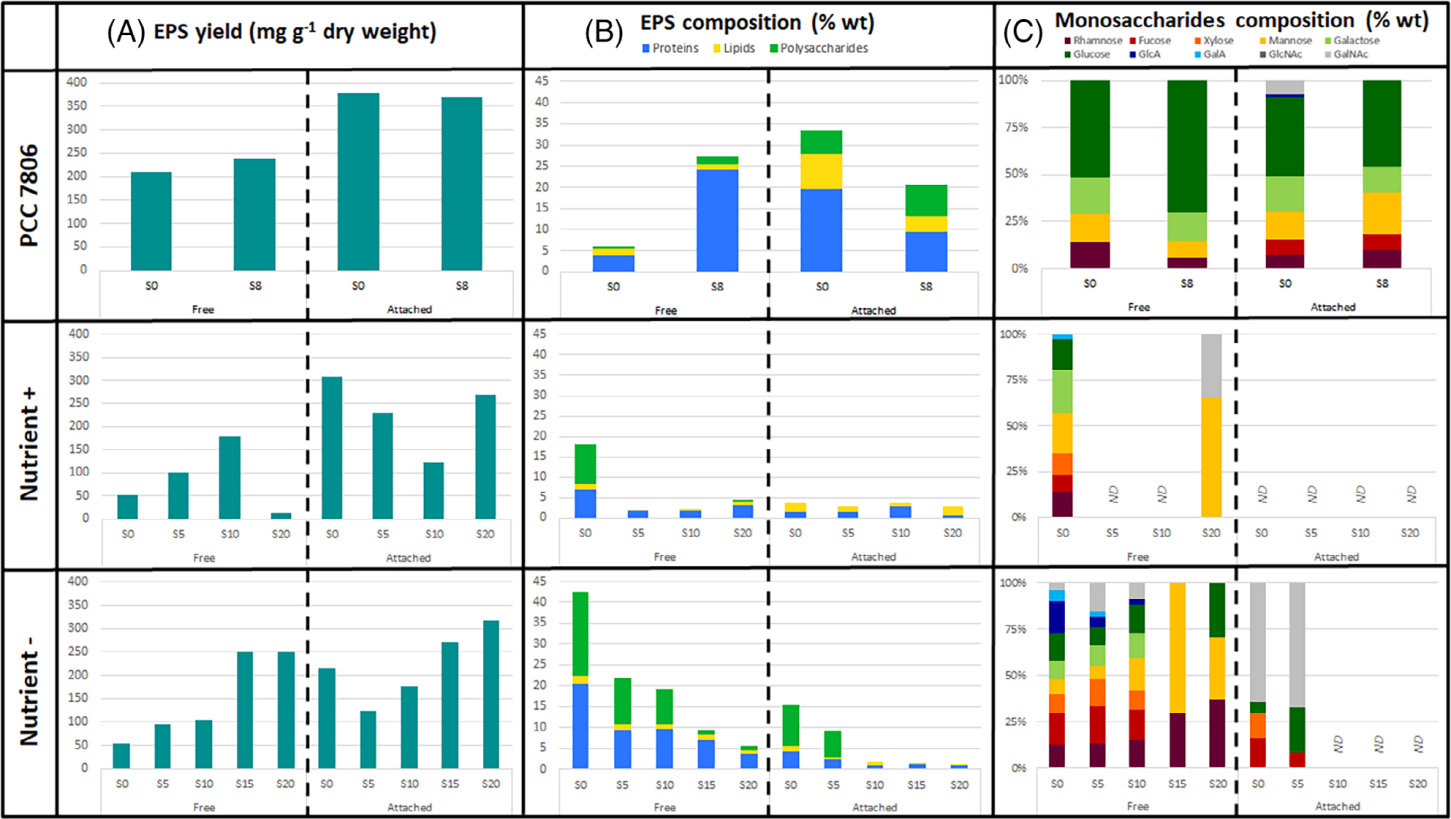
Free and attached EPS (A) yield, (B) proteins, lipids and polysaccharides composition and (C) monosaccharide composition of the polysaccharide fraction from the unicellular *M. aeruginosa* PCC 7806 strain and natural colonies of *Microcystis* at the end of the two batch experiments (Nutrient+ and Nutrient−) at salinity S = 0, S = 5, S = 10, S = 15 and S = 20. ND, not detected.

The total EPS yield produced by the unicellular PCC 7806 strain did not change at S = 8 compared to S = 0 culture condition (~ 550 mg g^−1^ dry weight; Figure 4A). Similarly, concerning the batch experiments, no marked change of the total EPS yield was observed at low and intermediate salinities compared to salinity 0 (~ 250 mg g^−1^ dry weight). Whereas at the highest salinities (S = 15 and 20) under nutrient‐limited condition, the total EPS were enhanced 2‐fold. However, at these high salinities, we cannot exclude that higher EPS production was partly due to cell lysis. To our knowledge, no previous study has reported the impact of increased salinity on the production of EPS from natural *Microcystis* colonies. The osmotic pressure created by the salinity may stimulate the production of EPS as demonstrated by Abbasi and Amiri (2008) on Gram‐negative bacteria. Hence, like other studies on the impact of abiotic stresses (temperature, light intensity, nutrients and metal ions) or biotic stresses (grazing, heterotrophic bacteria), salinity stress also promotes EPS production in *Microcystis*. EPS would act as a protective layer around the microorganism, helping it to retain moisture and prevent damage from the high levels of salt. Of note, the single cells *M. aeruginosa* strain produced as much EPS as natural colonies under combined stress condition (i.e., ~550 mg g^−1^ dry weight). Nevertheless, we did not observe colony formation by the PCC 7806 strain unlike what was observed for the unicellular *M. aeruginosa* PCC 7820 strain after its exposure to grazers (Yang et al., 2008).

An increase in salinity maintained a higher yield of attached EPS compared to free EPS in both the PCC 7806 strain and in the natural colonies of *Microcystis* regardless of the nutrient level (Figure 4A). More specifically, the yield of free EPS increased steadily with increased salinity (up to 5‐fold at S = 15 and 20 under nutrient‐limiting conditions compared to S = 0), while the yield of attached EPS first decreased at low salinities (S = 5 and 10) and increased at intermediate and high salinity levels. Hence, the highest yields of total EPS measured under combined stress conditions (S = 15 and 20 under nutrient‐limited condition) were mainly due to an overproduction of free EPS.

#### EPS composition

The total EPS composition of the unicellular PCC 7806 *M. aeruginosa* strain was globally characterized by a dominance of proteins (~ 30%), then polysaccharides and lipids representing less than 10% each but mostly found in the attached fractions whatever the salinity (Figure 4B). Of note, changes in the composition of EPS in response to higher salinity were mainly observed in the proportion of proteins of each EPS fractions. Hence, at S = 8, the proportions of proteins were higher in the free fraction (~25%) than the control (~5%) and the reverse was observed for the attached fractions (~20% and ~10% at S = 0 and 8, respectively). Therefore, the polysaccharide/protein ratio strongly decreased with increasing salinity conferring a hydrophobic character to free EPS (Santschi et al., 2020). The hydrophobic characteristics of EPS would promote the aggregation of cells between them, thus allowing a collective migration of cells on the surface of the water forming scums (Dervaux et al., 2015), an obligatory step before a bloom of *Microcystis*. The profile of monosaccharide composition in both free and attached EPS did not change with a moderate increase in salinity (Figure 4C). Similarly, Yang and Kong (2012) demonstrated no difference in monosaccharide composition of EPS from the PCC 7806 strain subjected to a grazing pressure. Nevertheless, HPSEC‐MALS analyses demonstrated a major modification of the molecular weight of free extracellular polysaccharides according to a moderate increase in salinity (Figure S2). Two populations of polysaccharides were always found in the free fraction with 20% of HMW polysaccharides (1.8 × 10^7^ g mol^−1^) and the appearance of more than 80% of MMW polysaccharides (1.7 × 10^5^ g mol^−1^). The effects of salinity may stimulate the production of MMW polysaccharides in free EPS of PCC 7806 following osmotic stress. Whereas the increase in salinity did not affect the proportion of the three populations of polysaccharides found in the attached fraction of the EPS.

Concerning the EPS produced by natural *Microcystis* colonies during the batch experiments, their composition in proteins and polysaccharides were highly altered under increased salinities for all samples (Figure 4B). In both experiments, the proportions of proteins and polysaccharides decreased with the increasing salinity and did not represent more than 5% of the weight of the EPS, or were no longer detectable by the methods used. Since the sampling of EPS occurred after 6–9 days of the experiment, it is likely that the composition of the EPS was modified, for example, by bacterial activity. Indeed, the heterotrophic bacterial community embedded in the cyanobacterial mucilage may degrade EPS components, including proteins and polysaccharides, especially under nutrient limitation, and use them as an energy source. Nevertheless, the polysaccharide/protein ratio strongly decreased with increasing salinity that may confer a hydrophobic character to natural *Microcystis* EPS independent of nutrient stress (Santschi et al., 2020). The relative hydrophobicity of EPS molecules is mainly attributed to proteins that will be involved in cell surface attachment, but also in the stabilization of the mucilaginous matrix and the development of a three‐dimensional mucilaginous architecture (Xu et al., 2011). On the other hand, lipids always represented only a small proportion (<3% wt) in free and attached *Microcystis* EPS and remained stable regardless of nutrient or salt stress. According to literature, lipids are not essential structural components of EPS and their relatively stable chemical structure makes them resistant to degradation under various environmental stresses, including nutrient or salt stress. This stability is due to the presence of non‐polar hydrocarbon chains in lipid molecules, which make them less reactive than other EPS components (Kehr & Dittmann, 2015).

Finally, monosaccharide composition was inferred in samples containing polysaccharides (Figure 4C), that is, retrieved mainly in free EPS under nutrient stress conditions (Figure 4B, Nutrient−). In the natural colonies of *Microcystis*, increased salinities led to a lower diversity of monosaccharides. Rhamnose, mannose and glucose mainly composed the free polysaccharides at salinities between 15 and 20 under nutrient stress conditions, while 9 out the 10 targeted monosaccharides composed the free EPS at lower salinities. Of note, attached EPS were mainly composed of *N*‐acetyl‐galactosamine with some minor amounts of rhamnose, xylose and glucose. Furthermore, HPSEC‐MALS results demonstrated a major difference in the molecular weight of free extracellular polysaccharides *Microcystis* colonies exposed to an increase in salinity (Figure S2A,B). Under an intermediate salinity stress from S = 5 to S = 15, the polysaccharides of MMW (from 5.7 × 10^4^ to 1.41 × 10^5^ g mol^−1^) dominated the population and contributed between ~60% and 100% of total polysaccharides. In parallel, the polysaccharides of HMW (from 6.3 × 10^5^ to 1.1 × 10^6^ g mol^−1^) decreased following an increase in salinity. Nevertheless, at high salinity (S = 20, Nutrient−), the proportion of these two populations of polysaccharides was reversed, and HMW polysaccharides became the majority. Consequently, an increase in salinity mainly caused the presence/production of high and medium molecular weight polysaccharides in the free EPS of the natural colonies of *Microcystis* under nutrient‐stress conditions. We again show here that natural colonies have higher proportions of high molecular weights than the unicellular strain, suggesting more complex biopolymers likely involved in colonies formation/cell aggregation. Moreover, the tendency to higher molecular weight is increased under stress conditions, suggesting a denser mucilage protecting the cells against abiotic stresses (salinity and nutrient limitation). While Kehr and Dittmann (2015) show that functional assignments of cyanobacterial exopolysaccharides are linked to their physico‐chemical properties, little is known about their biological significance. Rossi and De Philippis (2015) showed that in cyanobacteria the synthesis of exopolysaccharides contributes to a chemical/physical protection against abiotic and biotic stress factors. They suggest that polysaccharides with different molecular dimensions or composed of monosaccharides with specific properties (i.e., hydrophobicity, negative charge, etc.) protect to different extents the cells from harmful environments. Therefore, the cyanobacteria able to modulate these characteristics may have an ecological advantage and better thrive in harsh conditions.

## CONCLUSION

This study allowed for the first time to describe the production and the composition of both free and attached EPS from natural colonies of *Microcystis* under nutrient and salinity stress, and to compare with a unicellular *M. aeruginosa* strain (PCC 7806). As expected, the production of total EPS was higher for the mucilaginous colonial form than the unicellular one with a greater quantity of EPS in the attached fraction than in the free one. EPS produced by the colonial form were characterized by high molecular weight polysaccharides which, in contrast to the unicellular strain, were enriched in uronic acids and hexosamines that additionally confer the capacity to the cells to aggregate. In response to salinity or combined salinity and nutrient stress, we observed a higher production of EPS of the colonial form than the unicellular one. Of note, the free fraction was enriched of polysaccharides of high molecular weight which may confer protection to the colonial population against increasing salinity. Interestingly, our study revealed that other *Microcystis* species, here *M. wesenbergii*, are at least as salt‐tolerant as the well‐studied *M. aeruginosa*. Therefore, further studies on the characterization and production of EPS from different *Microcystis* species, if possible in colonial form, could definitely improve our understanding of the mechanisms involved in the formation and maintenance of the mucilaginous *Microcystis* bloom under various environmental conditions.

## AUTHOR CONTRIBUTIONS

**Oceane Reignier:** Formal analysis (lead); methodology (equal); visualization (lead); writing – original draft (lead); writing – review and editing (lead). **Myriam Bormans:** Conceptualization (lead); formal analysis (equal); methodology (equal); supervision (lead); validation (lead); writing – original draft (lead); writing – review and editing (lead). **Laetitia Marchand:** Formal analysis (equal); methodology (equal); writing – review and editing (equal). **Corinne Sinquin:** Formal analysis (equal); methodology (equal); writing – review and editing (equal). **Zouher Amzil:** Conceptualization (equal); funding acquisition (equal); writing – review and editing (equal). **Agata Zykwinska:** Formal analysis (equal); methodology (equal); validation (equal); writing – review and editing (equal). **Enora Briand:** Conceptualization (lead); formal analysis (equal); funding acquisition (lead); methodology (equal); project administration (lead); supervision (lead); validation (lead); writing – original draft (lead); writing – review and editing (lead).

## CONFLICT OF INTEREST STATEMENT

The authors declare no conflict of interest.

## Supporting information


**Figure S1.** *Microcystis* polysaccharide characterization with the mass fraction (%) (A) and the molecular weight, Mw (g mol^−1^) (B) of free and attached polysaccharides of the unicellular *M. aeruginosa* PCC 7806 strain and natural colonies of *Microcystis* during bloom and post‐bloom at salinity zero (HMW, high molecular weight; MMW, medium molecular weight; LMW, low molecular weight).

**Figure S2.** *Microcystis* polysaccharide characterization with the mass fraction (%) (A) and the molecular weight, Mw (g mol^−1^) (B) of free and attached polysaccharides (HMW, high molecular weight; MMW, medium molecular weight; LMW, low molecular weight) of the unicellular *M. aeruginosa* PCC 7806 strain and natural colonies of *Microcystis* at the end of the two batch experiments (Nutrient+ and Nutrient−) at salinity S = 0, S = 5, S = 10, S = 15 and S = 20. ND, not detected.

## Data Availability

The data that support the findings of this study are available from the corresponding author upon reasonable request.
