# Supplementary figures and images for: Production and composition of extracellular polymeric substances by a unicellular strain and natural colonies of Microcystis: Impact of salinity and nutrient stress
Source: Environ Microbiol Rep. 2023 Sep 11;15(6):783–96. doi: 10.1111/1758-2229.13200 (PMC10667651; doi:10.1111/1758-2229.13200)

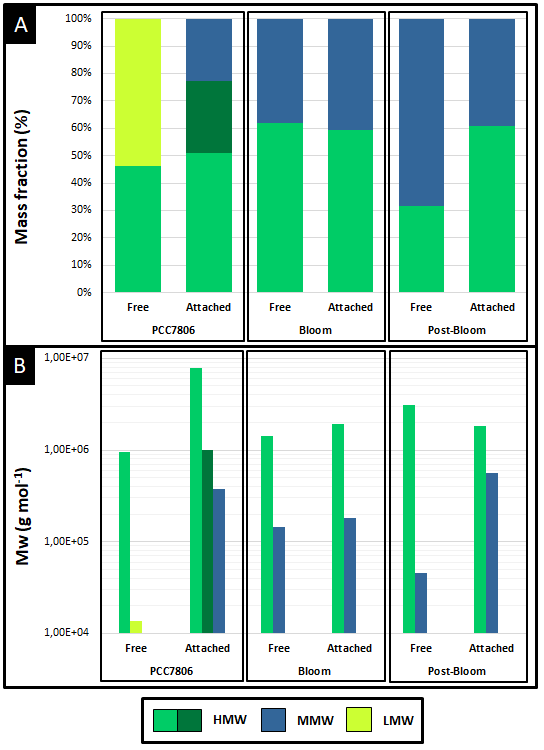

Supplement: Supplementary file 1 — Figure S1. Microcystis polysaccharide characterization with the mass fraction (%) (A) and the molecular weight, Mw (g mol−1) (B) of free and attached polysaccharides of the unicellular M. aeruginosa PCC 7806 strain and natural colonies of Microcystis during bloom and post‐bloom at salinity zero (HMW, high molecular weight; MMW, medium molecular weight; LMW, low molecular weight). [file EMI4-15-783-s002.png]

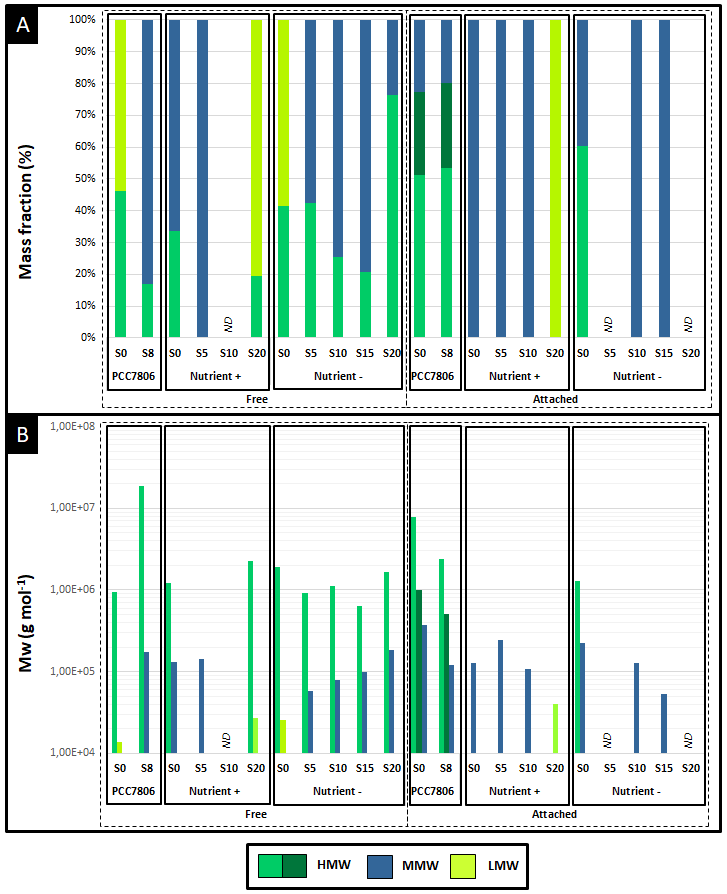

Supplement: Supplementary file 2 — Figure S2. Microcystis polysaccharide characterization with the mass fraction (%) (A) and the molecular weight, Mw (g mol−1) (B) of free and attached polysaccharides (HMW, high molecular weight; MMW, medium molecular weight; LMW, low molecular weight) of the unicellular M. aeruginosa PCC 7806 strain and natural colonies of Microcystis at the end of the two batch experiments (Nutrient+ and Nutrient−) at salinity S = 0, S = 5, S = 10, S = 15 and S = 20. ND, not detected. [file EMI4-15-783-s001.png]
